# Scoping Review of the Biomedical Investigations of Cellulose Nanocrystal-Based Hydrogels: A Critical Analysis of Current Evidence, Research Gaps and Future Perspectives

**DOI:** 10.3390/gels12030207

**Published:** 2026-02-28

**Authors:** Dinuki M. Seneviratne, Eliza J. Whiteside, Louisa C. E. Windus, Paulomi (Polly) Burey, Raelene Ward, Pratheep K. Annamalai

**Affiliations:** 1 School of Health and Medical Sciences, University of Southern Queensland, Toowoomba, QLD 4350, Australia; eliza.whiteside@unisq.edu.au (E.J.W.); louisa.windus@unisq.edu.au (L.C.E.W.); 2 School of Science, Engineering and Digital Technologies, Centre for Future Materials, University of Southern Queensland, Toowoomba, QLD 4350, Australia; polly.burey@unisq.edu.au; 3 Rural Clinical School, The University of Queensland, Toowoomba, QLD 4350, Australia; 4 School of Agriculture and Environmental Science, Centre for Future Materials, University of Southern Queensland, Toowoomba, QLD 4350, Australia; 5 First Nations Engagement, Institute for Resilient Regions, University of Southern Queensland, Toowoomba, QLD 4350, Australia; raelene.ward@unisq.edu.au; 6 School of Agriculture and Food Sustainability, University of Queensland, Brisbane, QLD 4072, Australia

**Keywords:** cellulose nanocrystals, hydrogel, biomedical models, healthcare applications, cell lines, animal models

## Abstract

Hydrogel-based products are used in many areas of biomedicine and healthcare. Recently, the incorporation of cellulose nanocrystals (CNC), a renewable and functional nanomaterial, into hydrogels has enhanced their functionality, particularly by imparting mechanical strength and structural integrity. This scoping review aims to appraise the types of biomedical models and assays that have been utilised to investigate the effects of CNC incorporation into hydrogels in tissue engineering, wound healing, medical implantation and drug delivery applications, and reports on the rationale for including these models and assays. A structured literature search was undertaken in major scientific databases (PubMed Central, PubMed, BioMed Central, ScienceDirect, Wiley and EBSCOhost), focusing on identifying primary research published between 2016 and 2024. From this process, fifteen studies providing biomedical analyses met the inclusion criteria. Most of these investigations employed in vitro cell-line models (*n* = 12), with a smaller number utilising in vivo experimental systems (*n* = 5). Across the included studies, CNC incorporation typically yielded measurable performance gains: reported compressive or storage modulus improvements of 20–40% over hydrogel-only controls, consistently high cell viability (>85%) across multiple human and murine cell types for up to 21 days, and sustained drug release profiles (days–weeks) in stent and antitumour contexts. Where quantified, functional outcomes in vivo included preserved graft volume (autologous fat grafts) and reduced intimal hyperplasia signals in vascular graft models. Critical gaps included heterogeneous CNC sources and surface chemistries, inconsistent reporting of CNC concentration and hydrogel formulation parameters, the limited duration and scope of biocompatibility testing, and minimal alignment with standard evaluation protocols, constraining reproducibility and cross-study comparability. To date, there are no human clinical trials of CNC-hydrogels. Translational readiness will require standardised ISO-compliant biocompatibility evaluations. Large-animal studies under relevant mechanical and physiological conditions, and rigorous long-term degradation and immunogenicity assessments to de-risk progression to human trials. We recommend standardised CNC sources and surface functionalisation reporting, concentration (wt%) ranges, hydrogel rheological characterisation (G′, G″, swelling), and consistent biological endpoints (viability, differentiation, inflammation panels) to enable robust meta-analyses and translational benchmarking. Distinct from prior nanocellulose reviews that emphasise material synthesis and properties, this analysis centres on the biomedical models and assays applied to CNC-incorporated hydrogels, identifying the methodological convergence and divergence that directly impact translational pathways.

## 1. Introduction

Hydrogels are three-dimensional polymeric networks that demonstrate water-retention capabilities while maintaining structural integrity. The other properties of hydrogels, including biocompatibility, tuneable mechanical properties, and the ability to mimic the extracellular matrix, have resulted in hydrogels being developed and commercialised for a wide range of biomedical and healthcare applications [1]. The most common applications are in drug delivery systems, biosensors, and tissue engineering [2,3]. Researchers have recently developed and investigated hydrogels combined with cellulose-derived nanomaterials, such as cellulose nanocrystals (CNC), also known as cellulose nanowhiskers (CNW) or nanocrystalline cellulose (NCC). Owing to their unique combination of nanoscale geometry, surface chemistry, mechanical reinforcement capability [4], and biocompatibility [5], these rod-like/whisker-like nanomaterials are utilised in the fabrication of hydrogels. CNC can be derived from a wide range of natural sources of cellulose, such as cotton and rice husk, and their characteristics can be functionalised when incorporated into hydrogels [6]. The addition of CNC derived from the majority of plant biomass provides greater mechanical strength and tuneable swelling behaviour, and biodegradability to nanocomposite hydrogels, when compared to hydrogels alone [7].

In addition to their enhanced strength, modifiable swelling behaviour, biodegradability and sustainability, CNC-incorporated hydrogels can also be designed to enhance adhesion to various surfaces, including cells and tissues [8], improve cell proliferation and/or migration capabilities [9,10], and increase drug encapsulation for sustained drug release in the body [11]. Several studies have reported the utility/potential of CNC in enhancing the performance of hydrogels for biomedical engineering and healthcare applications [12,13]. Despite these promising attributes, current research lacks a unified experimental framework. Studies vary widely in cell line selection, assay endpoints, and animal models, limiting comparability and reproducibility. Furthermore, the absence of human trials raises questions about translational readiness [13,14]. Hence, a more extensive understanding of the effects of CNC-incorporated hydrogels in relevant biological environments is still required before they are utilised in clinical applications. This highlights the importance of in vitro and in vivo biomedical models as they allow for evaluation of the interactions between CNC-incorporated hydrogels and cells, tissues, and other physiologically relevant materials to assess the compatibility, efficacy, and functionality of the hydrogels for a specific healthcare or biomedical application.

Preclinical biocompatibility studies commonly employ two-dimensional or three-dimensional in vitro cell culture studies using commercially available primary and/or secondary cell lines, and/or in vivo studies using animal models. These models have also been used to assess the biocompatibility of CNC-incorporated hydrogels, as well as cellular responses and cell–material interactions within the studied microenvironment [15]. Once there sufficient positive results have been obtained using in vitro and in vivo studies, potential biomedical and healthcare applications are assessed using human clinical trials [16]. In the current literature landscape, such detailed biomedical investigations are limited. Hence, this scoping review aims not only to map existing evidence but also to critically analyse methodological gaps and propose strategies for harmonising the biomedical testing of CNC hydrogels.

This scoping review collated and analysed the biomedical models and testing methods used in published research on CNC-incorporated hydrogels for biomedical and/or healthcare applications. The rationale for conducting this scoping review was to determine the current status of research and whether human clinical trials have been undertaken, and to inform future researchers on the appropriate biomedical models and testing methods to evaluate CNC-based hydrogels for potential biomedical and healthcare applications. By critically mapping the experimental frameworks across applications, this scoping review identifies translational bottlenecks and proposes a roadmap for future research to bridge the gap between laboratory findings and clinical implementation.

## 2. Results and Discussion

**Figure 1:**
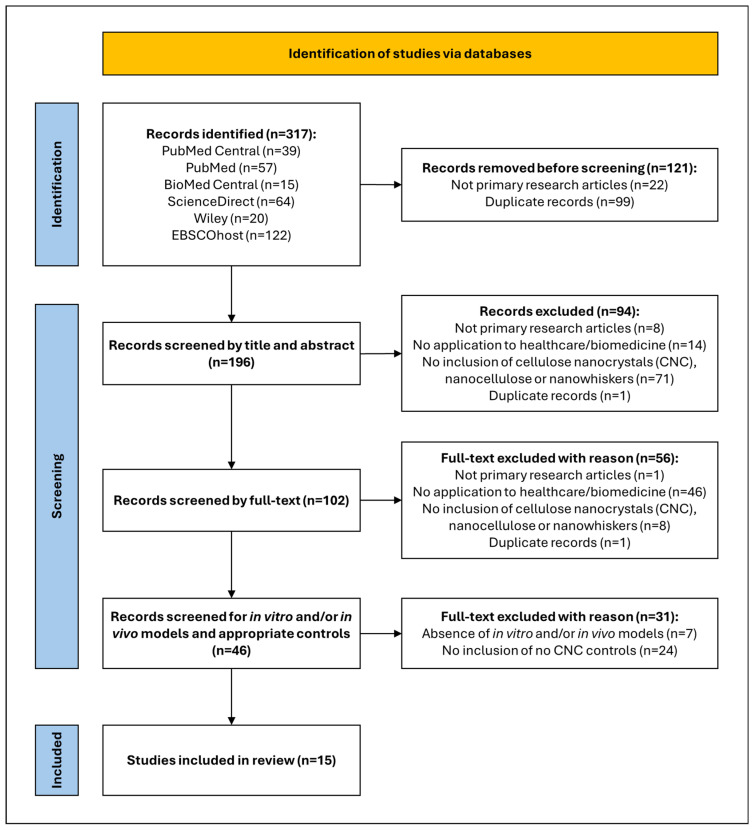
PRISMA flow chart of the literature search strategy [13,17].

The literature review search across the six aforementioned electronic databases obtained 317 primary research articles, of which 15 articles were included after conducting the screening process detailed in Figure 1. Many of the 315 primary research articles report descriptive approaches without rigorous hypothesis-testing or comparative controls, which limit confidence in the observed biocompatibility outcomes. The PRISMA flow (Figure 1) shows a high exclusion rate, primarily due to the lack of CNC incorporation, the absence of biomedical models, or a lack of hydrogel-only controls. This indicates that while CNC–hydrogel synthesis is widespread, integrative experimental designs that directly test biological performance versus appropriate controls remain scarce, highlighting a gap that hinders the synthesis and translation of evidence.

**Figure 2:**
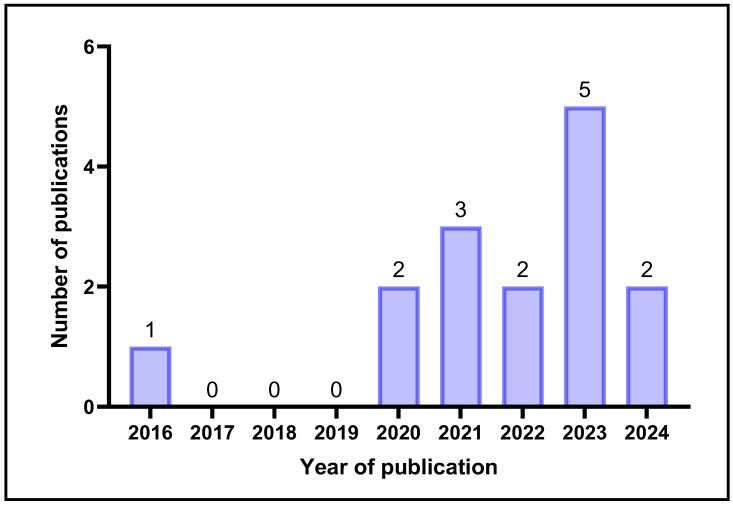
Publication years of the included studies and the number of studies published each year (created by D.M. Seneviratne using GraphPad Prism 10.5.0).

Of the 15 included studies, 14 (approximately 93.33%) were published in 2020–2024, compared with 1 (approximately 6.67%) in 2016–2019, indicating a marked increase in biologically tested CNC–hydrogel investigations in the most recent five-year period, as displayed in Figure 2. While there were no in vitro or in vivo model-based studies conducted from 2017 to 2019, the highest number of studies on cell- and animal-based models (*n* = 5) was conducted in 2023. The acceleration in 2020–2024 plausibly reflects multiple converging drivers: 1. The increased prioritisation of renewable biomaterials in biomedicine; 2. The improvement in CNC isolation and functionalisation methods, yielding reproducible, higher-yield materials; and 3. The diffusion of biofabrication technologies (e.g., extrusion/bioprinting) that benefit from the rheology-modulating and reinforcement effects of CNC. These ecosystem changes likely lowered barriers to deploying CNC within biologically relevant hydrogel platforms. The 2017–2019 period likely reflects a period where CNC research prioritised materials synthesis, characterisation, and non-biomedical applications, with constrained availability of biomedical-grade CNC and unresolved dispersion/sterility challenges for cell-compatibility formulations. Consequently, model-based biocompatibility studies lagged until fabrication workflows and quality controls improved.

**Figure 3:**
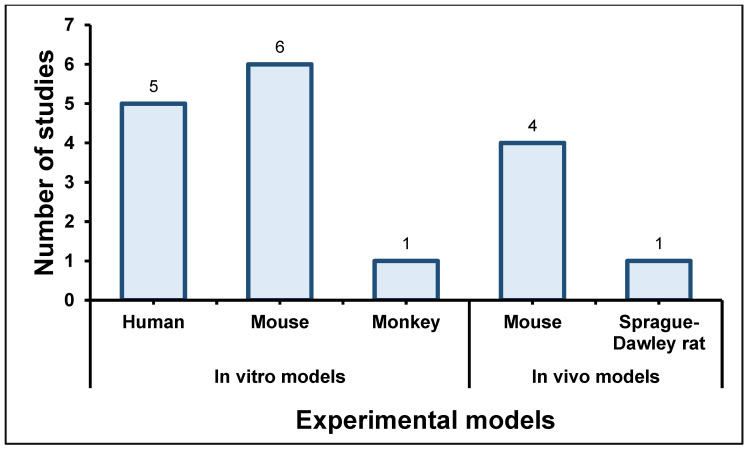
The distribution of in vitro and in vivo models used in the included studies (created by D.M. Seneviratne using GraphPad Prism 10.5.0).

Categorisation by application shows the concentration of in vitro-only studies in tissue engineering and drug delivery, with medical implant investigations most frequently combining in vitro and in vivo endpoints. The majority of the studies utilised in vitro models of human (*n* = 5), mouse (*n* = 6) and monkey (*n* = 1) cell lines, while in vivo models included either mice (*n* = 4) or Sprague-Dawley rats (*n* = 1), as displayed in Figure 3. There were no published studies on clinical trials of CNC-incorporated hydrogels. Therefore, it can be stated that in vitro models had a 70.59% (*n* = 12) probability of being utilised as experimental models, compared to the in vivo models, which were utilised at a 29.41% probability (*n* = 5).

The included studies show that most hydrogels incorporated CNC in the 0.5–5 wt% range (where reported), with chitosan/alginate/GelMA/gelatin as the recurrent base polymers. The most consistently reported endpoints were short-term viability (>85–95%), osteogenic and chondrogenic differentiation markers (ALP, collagen, sGAGs), and mechanical reinforcement (increased G′/compressive modulus). Drug delivery studies reported sustained release spanning days to weeks; in vivo studies emphasised graft retention, vascular patency, and reduced inflammatory signatures. The reporting of CNC surface chemistry and dispersibility was inconsistent, limiting precise estimates of cross-study effects. The characterisations of CNC-incorporated hydrogels, including physicochemical properties and biological endpoints, in the included studies are summarised in Table 1. Speculative statements are based on observed trends and should be interpreted cautiously.

### 2.1. Tissue Engineering Applications

CNC-based hydrogels have been explored as promising biomaterials for biomedical engineering applications, such as three-dimensional scaffolds for bone and cartilage tissue engineering. The various biomedical models and assays utilised by the included studies to investigate the biocompatibility and biological capabilities of CNC-based hydrogels in tissue engineering for bone or cartilage repair or reconstruction are summarised in Table 2.

The main finding from Table 2 was that the majority of studies used primary cell lines derived from human or mouse tissue; however, no investigation involving in vivo (animal) models was reported for CNC–hydrogels intended for bone or cartilage tissue engineering applications. This limitation (i.e., omitting animal studies) is critical because bone and cartilage regeneration require load-bearing assessments under physiological conditions, which cannot be replicated in static cell culture systems. Nevertheless, the structural integrity and mechanical strength imparted by CNC in hydrogels were shown to be promising biomaterials for bone tissue engineering applications. All three of the included studies investigated the CNC-based hydrogels’ abilities in terms of bone repair and regeneration using the MC3T3-E1 mouse osteoblast cell line model [18,19,20]; two of these studies were conducted in the same laboratory [18,19]. In addition to measuring cell viability and proliferation, osteogenic differentiation was measured by quantifying alkaline phosphatase (ALP) activity, calcium deposition and collagen formation. Osteoblasts expressed high levels of ALP during early differentiation, before bone matrix mineralisation [31], indicating early osteogenic differentiation [18,19,20]. During osteogenesis, calcium ion accumulation contributes to matrix mineralisation, as calcium is a crucial component of bone. Two studies quantified calcium deposition using von Kossa staining [18,19]. Another study used scanning electron microscopy with energy-dispersive X-ray spectroscopy to observe apatite layer formation and its distribution on hydrogels in simulated body fluids [20]. As a primary component of the extracellular matrix (ECM), the formation of collagen fibres indicates the production of a collagen-rich ECM during the osteogenic process. The extent of collagen formation promoted by CNC-based hydrogels was monitored via haematoxylin and eosin (H&E) staining [18,19].

**Figure 4:**
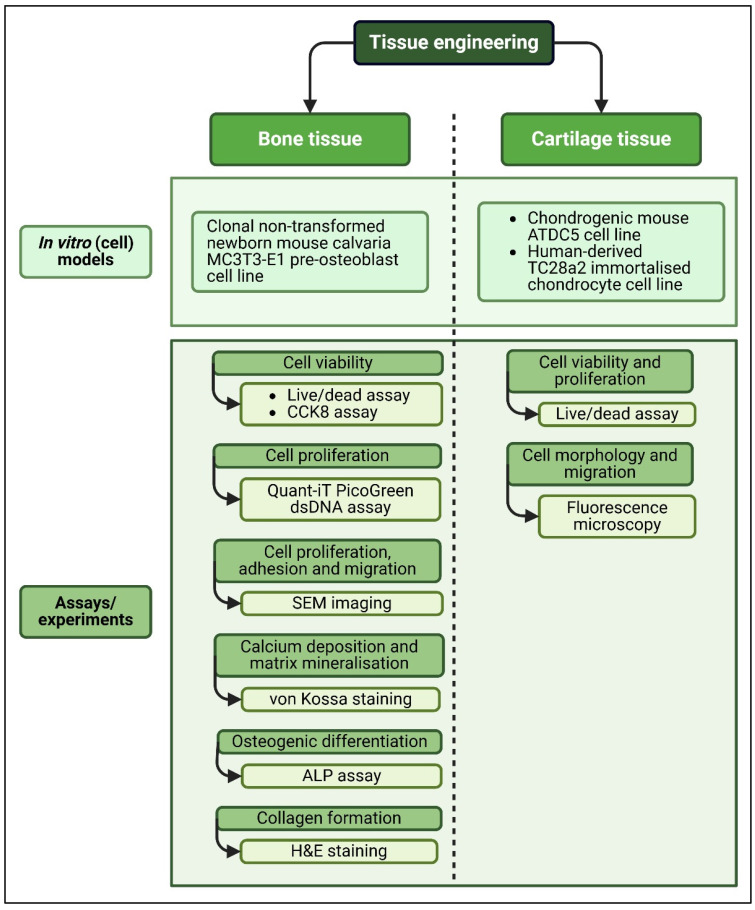
Summary of bone and cartilage tissue engineering applications of the included studies and the effects of CNC incorporation (created by D.M. Seneviratne using BioRender (https://app.biorender.com/ accessed on 14 September 2025)).

CNC-based hydrogels have demonstrated tuneable viscoelastic and load-bearing properties that are favourable for cartilage tissue engineering. The included studies utilised in vitro models based on either mouse ATDC5 [21] or human TC28a2 [22] Chondrocyte cell lines to study the cartilage regeneration ability of CNC-based hydrogels. Laser scanning confocal microscopy and fluorescence microscopy images of live/dead assays with calcein AM/propidium iodide and ethidium homodimer staining, respectively, were used to measure chondrocyte viability [21,22], while cell morphology and migration were observed by fluorescence microscopy in one study [22]. These findings are summarised in Figure 4.

### 2.2. Wound Healing and Repair

Only one study investigated CNC-incorporated hydrogels, demonstrating biocompatibility for potential wound dressing applications [23] (see Table 3).

This study utilised human dermal fibroblasts to assess biocompatibility as cell viability over 7 days in response to a CNC-based hydrogel using a resazurin staining method. Because microbial burden hinders wound healing, this study also investigated the antimicrobial activity of the CNC-based hydrogel using a 24 h agar disc diffusion method. The microbes that were used included *Escherichia coli* and *Staphylococcus aureus*, both common wound-associated bacteria. [23]. The extent of the inhibition halo or cleared zone surrounding the CNC-hydrogel disc represents a direct inhibition of bacterial growth. There may also be another halo further away from the disc, and this may indicate the inhibition of bacterial growth without necessarily killing the bacteria, demonstrating a bacteriostatic effect [23]. The reliance on a single study for wound healing applications indicates a significant research gap. Furthermore, antimicrobial testing via disc diffusion lacks clinical relevance, as it does not mimic wound microenvironments or biofilm formation.

### 2.3. Medical Implants

The potential of CNC-based hydrogels for use in various medical implants (excluding tissue engineering) was investigated in three of the included studies. The biomedical models and experiments that were used in testing these CNC-incorporated hydrogels are outlined in Table 4.

The studies included investigating the applicability of CNC-incorporated hydrogels in autologous fat grafting [24], the prevention of coronary stent restenosis [25] and cardiac valve regeneration [9] using a mixture of in vitro (cell lines) and in vivo (animal) models. An in vivo Balb/C mouse model was employed to examine the effects of CNC-based hydrogels in autologous fat grafting, focusing on whether the addition of CNC reduced the resorption of grafted tissue. The maintenance of adipocyte volume, content, and characteristics after 30 days post-graft was macroscopically assessed by observing the explanted hydrogel grafts and qualitatively assessed through haematoxylin and eosin (H&E) staining of tissue section [24].

Restenosis after coronary artery bypass grafting and haemodialysis access is a major issue with using vein grafts. Restenosis is primarily promoted through endothelial–to-mesenchymal transition (EndMT), whereas the autophagy cellular degradation pathway has been shown to mitigate the risk of restenosis. Due to the reported anti-inflammatory activities of CNC, one of the included studies investigated the ability of CNC-based hydrogels to prevent EndMT via autophagy [25]. To further optimise the outcomes, the authors incorporated astragaloside-IV, an anti-inflammatory drug that could also induce autophagy. They studied its release rates in an in vitro HUVEC human endothelial cell line model using liquid chromatography mass spectrometry and observed any induced morphological changes using bright field microscopy. Furthermore, the viability, proliferation, and DNA damage of the HUVEC cell line in response to the CNC–hydrogels were measured using a CCK8 assay, live/dead assay, and comet experiment, respectively. This study utilised a mouse model and Sprague-Dawley rats to investigate the biocompatibility of the stent and its ability to prevent the EndMT process. Immunohistochemistry on local tissues, along with haematoxylin and eosin (H&E) and Masson staining on mouse organs, was performed to assess compatibility and the potential damage induced by hydrogel-based stents. Blood and spleen samples were then tested for anti- and pro-inflammatory responses using flow cytometry (for lymphocytes, CD45, CD3, CD45R, CD11B+ F4/80+ and CD11B+), enzyme-linked immunosorbent assay (ELISA; for IL-1B, IL02, IL-6, IL-4, IL-10 and IL-13) and reverse transcription quantitative polymerase chain reaction (RT-qPCR; conducted on spleen samples for IL-1B, IL-2, IL-6, IL-4, IL-10 and IL-3). Similarly, RT-qPCR on spleen tissue and a vein graft section were conducted in the Sprague-Dawley rats (for IL-1B, IL-2, IL-6, IL-4, IL-10, IL-13, TGF-β, CD31, α-SMA, slug, snail 1, twist, vimentin, beclin 1, LC3II, p62, AMPK and mTOR), along with Western blotting on vein grafts (for α-SMA, CD31, vimentin, slug, snail, twist, LC3II, p62, beclin 1, AMPK, p-AMPK, mTOR and p-mTOR) to support that AS-IV retains its ability to inhibit EndMT even after its release from CNC-based hydrogel stents. Additionally, the authors utilised colour Doppler detection to measure blood flow rate through the grafted veins and haematoxylin and eosin (H&E) staining to evaluate any changes in blood vessel measurements that could indicate restenosis [25].

CNC-based hydrogels have been explored as a platform for cardiac valve regeneration; one of the included studies used an HADMSC cell line in vitro model to investigate these capabilities [9]. Similar to the majority of CNC–hydrogel biocompatibility studies, cell viability, migration and morphology were assessed using both the MTT and live/dead assays. Double-stranded DNA (dsDNA), sulphated glycosaminoglycan (sGAG) and hydroxyproline contents were measured via dsDNA assay, DMMB assay/Alcian blue staining and DMAB assay, respectively. While myofibroblastic activation and chondrogenic differentiation were desired in cardiac valve regeneration, osteogenic differentiation was not preferred, as it could lead to valve calcification. Alpha smooth muscle actin (α-SMA), vimentin, matrix metalloproteinase 1 (MMP1) and MMP2 expression levels were measured as indicators of myofibroblastic activation, while aggrecan (ACAN) and SRY-box transcription factor 9 (Sox9) expression levels indicated chondrogenic differentiation. Osteogenic differentiation was determined via Alizarin red staining and measuring the levels of α-SMA, vimentin, runt-related transcription factor 2 (Rnx2), osteocalcin (OCN) and osteopontin (OPN) expression [9]. None of the studies evaluated mechanical durability under dynamic physiological conditions, a prerequisite for cardiovascular or orthopaedic applications.

**Figure 5:**
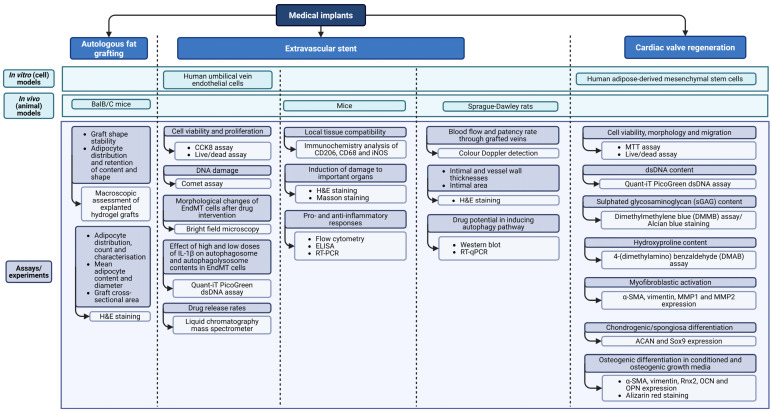
Summary of the included studies investigating CNC–hydrogels in medical implants (created by D.M. Seneviratne using BioRender (https://app.biorender.com/ accessed on 14 September 2025)).

It can be observed that the included studies have not utilised consistent in vivo models or testing assays when investigating the various medical implant applications of CNC-based hydrogels; however, the testing that was undertaken using animal models is comprehensive (Figure 5).

In autologous fat grafting, it is critical to evaluate the integration of CNC-based hydrogels with surrounding tissues and their biocompatibility by assessing host immune responses, angiogenesis and long-term graft retention. It is important to test vein grafts under normal physiological blood flow conditions to assess whether CNC-based hydrogel stents can withstand mechanical stress while also promoting vascular endothelialisation. Similarly, testing CNC-based hydrogels under dynamic cardiovascular conditions is essential for cardiac valve engineering. This may help to establish their mechanical durability to meet the biological demands of heart valves and determine their ability to resist calcification. Therefore, it is evident that these medical implant applications should be further tested using in vivo models before progressing into human clinical trials.

### 2.4. Drug Delivery Applications

The included studies utilised CNC-based hydrogels to load various drugs, primarily for antimicrobial and antitumour activities. Additionally, these hydrogels were used as soft robots for biomolecule delivery and as platforms to explore the drug delivery capabilities of loaded drugs. Additional information on these studies is summarised in Table 5.

**Figure 6:**
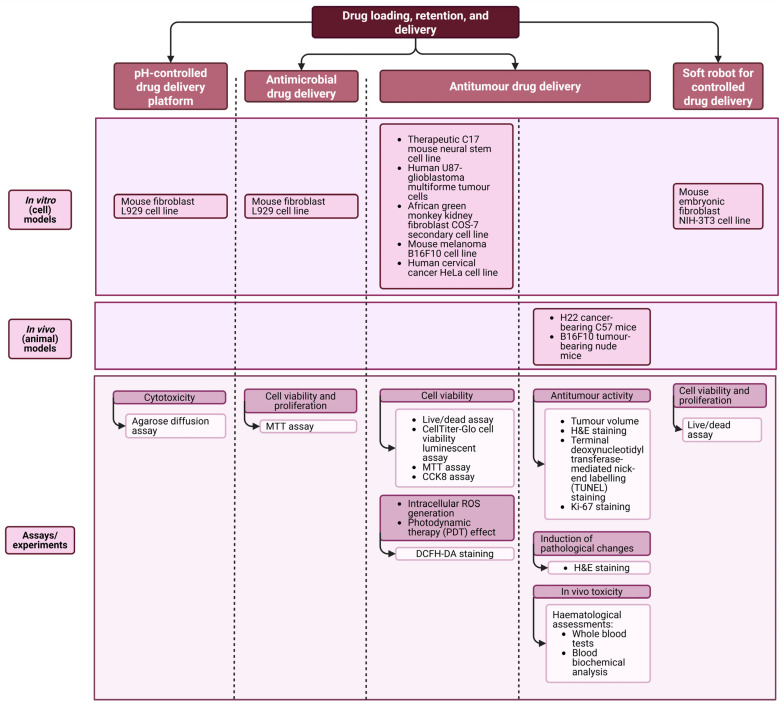
Summary of the drug loading, retention and delivery applications of the included studies (created by D.M. Seneviratne using BioRender (https://app.biorender.com/ accessed on 14 September 2025)).

The findings of Table 5 reveal that in vivo (animal) models have only been utilised to investigate the properties of drug-loaded CNC-based hydrogels in two studies to date [28,29], whereas the majority of the included studies utilised in vitro cell lines. These findings are summarised in Figure 6.

The effects of drug-incorporated CNC-based hydrogels on the cell viability of various in vitro models, such as therapeutic C17 mouse neural stem cells [27] and mouse embryonic fibroblast NIH-3T3 cells [30], were measured using live/dead assays, while MTT assays were also conducted using mouse fibroblast L929 cells [11] and African green monkey kidney fibroblast COS-7 cells [28]. One study used an agar diffusion assay to evaluate the changes within mouse L929 fibroblast layers and zones of fibroblast inhibition as indicators of cytotoxicity [26]. The tumour cell killing activities of TRAIL-protein- and nanocarbon dot-embedded CNC-based hydrogels on human U87-glioblastoma multiforme tumour cells, and mouse melanoma B16F10 cells and human cervical cancer HeLa cells were evaluated using CellTiter-Glo cell viability luminescent assay [27], CCK8 assay and live/dead assay [29], respectively. Additionally, DCFH-DA staining was utilised on B16F10 and HeLa cells to investigate whether CNC-based hydrogels induce intracellular reactive oxygen species (ROS) generation and can induce photodynamic therapy (PDT) effects [29].

Two studies conducted haematoxylin and eosin (H&E) staining on tumours and other critical organs to investigate the antitumour activity of doxorubicin- and carbon nanodot-loaded CNC-based hydrogels on H22 tumour-bearing C57 mice [28] and B16F10 tumour-bearing nude mice [29], respectively. Analysis of the tumour volume was utilised as an antitumour marker [28], where haematological assessments, including whole blood tests and biochemical analysis, and the staining of tumour slices using terminal deoxynucleotidyl transferase dUTP nick-end labelling (TUNEL) and ki-67 staining [29], were also conducted to explore the antitumour activity of doxorubicin- and carbon nanodot-loaded CNC-based hydrogels, respectively.

**Figure 7:**
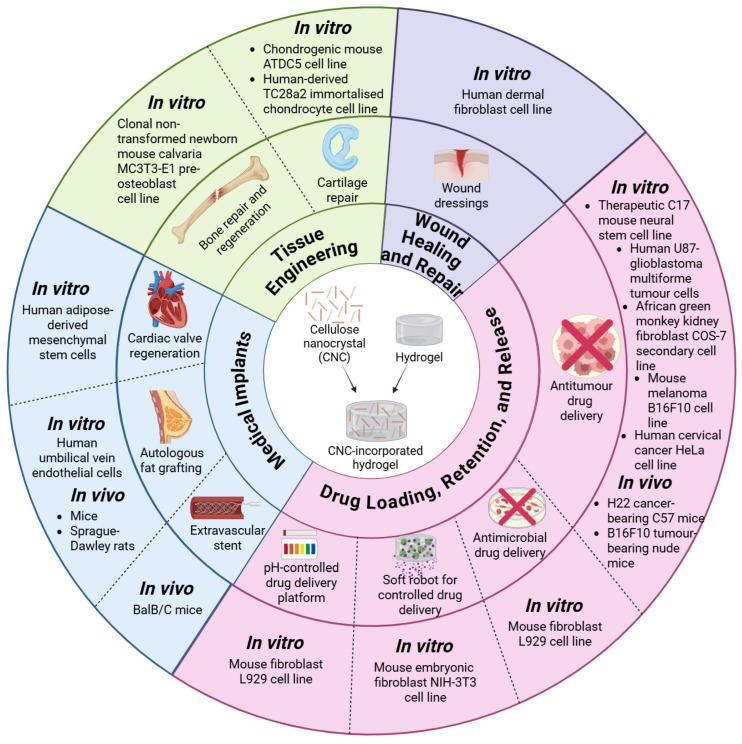
The in vivo and in vitro biomedical models utilised in investigating the biomedical applications of CNC-incorporated hydrogels, where each colour represents a distinct biomedical application domain, such as tissue engineering, wound repair, drug delivery and medical implants (created by D.M. Seneviratne using BioRender (https://app.biorender.com/ accessed on 14 September 2025)).

Overall, this literature review identified the biomedical models and assays/experiments that have been utilised by current research studies investigating the biomedical applications of CNC-incorporated hydrogels, while highlighting the minimal usage of in vivo models and the lack of human clinical trials in these research investigations. The research findings gathered through this literature review are summarised in Figure 7. The synthesis of the 15 studies collectively indicates that CNC incorporation 1. enhances mechanical resilience (higher G′/modulus, improved structural integrity), 2. maintains higher cytocompatibility across diverse mammalian cell lines over 1–21 days, and 3. augments functional performance in specific contexts, supporting adipocyte retention in grafts, modulating EndMT-related pathways with drug-loaded stents, and enabling antitumour effects through controlled release or ROS-mediated PDT. However, the durability of these effects beyond weeks, and their consistency across CNC sources and hydrogel chemistries remains to be established. The increase in endpoint sophistication over time is noteworthy: early studies focused on acute cytotoxicity and viability assays while later work probed lineage-specific differentiation (ALP, mineralisation; sGAGs), migration and morphology under load, and in vivo functional read-outs (graft retention, patency, tumour progression). This trajectory suggests growing confidence in CBC–hydrogel biocompatibility and a shift toward application-relevant performance metrics.

The included studies highlighted the variability regarding model quality and reproducibility. Few studies reported standardised CNC characterisation (particle size distributions, crystallinity index, sulphur content), batch-to-batch consistency, or sterility/pyrogen testing; biological protocols often omitted ISO-aligned biocompatibility panels (e.g., irritation/sensitisation), and mechanical assessments lacked standardised rheological protocols. We recommend adopting shared reporting checklists covering the CNC source/chemistry, dispersion metrology, hydrogel formulation parameters, and biological endpoints to permit reproducible cross-study comparisons. Furthermore, regulatory pathways for CNC-based biomaterials remain underdeveloped, necessitating early engagement with regulatory bodies to facilitate clinical translation.

Future studies on CNC-incorporated hydrogels should adopt a harmonised biomedical evaluation framework that includes a full physicochemical characterisation of CNCs (source, size, crystallinity, surface chemistry, endotoxin), transparent hydrogel formulation parameters, standardised rheology and swelling protocols, and ISO-aligned biological assays capturing viability, inflammation, and application-specific functional endpoints. In vivo investigations should incorporate randomisation, blinding, systemic toxicity panels, and physiologically relevant functional readouts. The reporting of sterilisation, endotoxin burden, and GMP-relevant processing is essential to support translational readiness. To strengthen structure–function understanding, comparative studies across CNC chemistries and concentrations should be conducted under matched hydrogel conditions, supported by advanced 3D models and organ-on-chip systems. The consistent implementation of these guidelines will significantly improve the reproducibility, comparability, and translational potential of CNC–hydrogel in biomedical research.

## 3. Conclusions

This scoping review critically analysed the biomedical models and assays used to evaluate CNC-incorporated hydrogels for biomedical and healthcare applications and assessed the relevance of these investigations for real-world applications. Where reported, CNC concentrations clustered between 0.5 and 5 wt%, with typical gains in storage/compressive modulus of 20–40% over hydrogel-only controls, consistently high cell viability >85% across diverse mammalian cell lines, sustained drug release over days to several weeks, and in vivo studies showing graft retention and antitumour effects versus controls. Across applications, short-term in vitro viability and lineage-appropriate differentiation assays reliably indicate biocompatibility and bifunctionality; however, translation-critical endpoints, including mechanical durability under physiological loading, immune modulation over weeks to months, and retained function in vivo, remain the decisive predictors of clinical promise. Studies that included in vitro tests and progressed to small animal in vivo experiments yielded the most decision-relevant insights, particularly in implant and antitumour delivery contexts.

Mechanistically, CNCs’ high aspect ratio and surface hydroxyl/sulphate groups promote hydrogen bonding and physical entanglement within polymer networks, increasing crosslink density and load-bearing capacity (higher G′/moduli) while modulating swelling and porosity. These changes may enhance cell–matrix interactions, including adhesion and proliferation, and enable controlled diffusion for drug release. Additionally, in stent platforms, they support anti-inflammatory drug delivery with reduced EndMT signatures. The lack of standardised protocols is noteworthy; adopting an ISO biological evaluation framework with consistent rheology/swelling protocols and pre-registered study designs would improve reproducibility and compatibility across CNC–hydrogel investigations and facilitate regulatory alignment. Nonetheless, several translational barriers persist, including variability in CNC source and surface chemistry (affecting dispersion, crosslinking, and biological response), limited sterility and endotoxin validation for clinical-grade materials, and insufficient long-term degradation and immunogenicity data, particularly under physiological loading conditions. The absence of large-animal studies and good manufacturing practice (GMP)-compatible fabrication workflows further constrains the bench-to-bedside pathway. Addressing these gaps will require coordinated efforts to standardise material specifications, validate biological performance across model tiers, and integrate regulatory considerations early in scaffold design.

Clinical Relevance and Likely Near-Term Opportunities: Although the field is in its infancy and no clinical trials of CNC-incorporated hydrogels were identified, the current evidence suggests several applications that are closest to clinical feasibility. These include the following: 1. vascular support applications (e.g., external stents) where combined in vitro/in vivo data already demonstrate biocompatibility and pathway-level effects; 2. adipose tissue graft retention with in vivo volume retention signals; and 3. injectable antitumour depots enabling sustained chemotherapeutic or photo-therapeutic delivery with efficacy in murine models. Progress in these indications will depend on the development of ISO-aligned biocompatibility packages, animal/in vivo durability and patency studies under physiological loading, and GMP-ready processing with batch-release criteria (including sterility and endotoxin limits).

To convert promising prototypes into translational candidates, we propose the following roadmap for future research:

(1) The integration of advanced 3D tissue models and organ-on-chip systems to generate application-relevant functional readouts (barrier integrity, contractility, vascular flow) that better predict in vivo performance.

(2) The systematic evaluation of long-term biodegradation, calcification, and local/systemic immune responses under physiologically relevant mechanical regimes, which can be extended to large animal studies where applicable.

(3) Comparative assessments of CNC against other nanocellulose forms (e.g., cellulose nanofiber and bacterial nanocellulose) and surface-functionalised CNC within matched hydrogel formulations to isolate structure–function relationships.

(4) Standardised reporting, with the mandatory disclosure of CNC source and surface chemistry, dispersion metrics (size distributions, crystallinity, surface groups), hydrogel rheology (G′/G″ under defined strain/frequency), swelling/porosity, and harmonised biological endpoints to enable meta-analyses.

(5) Translation-oriented workflows: pre-registered protocols, ISO 10993-aligned test batteries, stability and shelf-life studies, and GMP-compatible fabrication with validated sterilisation and endotoxin control [32].

Taken together, CNC hydrogels emerge as bioactive, sustainable and mechanically tuneable composites whose performance can be engineered through the precise control of CNC chemistry, loading, and network architecture for next-generation biomedical devices. Realising this potential will require broader interdisciplinary collaboration between materials scientists, biomedical engineers, and clinicians to co-design clinically meaningful endpoints, align models with their intended use, and accelerate the preclinical-to-clinical transition.

In summary, while materials development and in vitro cell-based studies are expanding, in vivo evidence remains limited for most applications, constraining immediate translation. The targeted roadmap and standardisation recommendations presented above provide practical steps to strengthen the quality of evidence, improve cross-study comparability and reduce the risk in the progression towards clinical trial evaluations of CNC–hydrogel platforms.

## 4. Methodology

### 4.1. Protocol

The literature search was conducted in accordance with the Preferred Reporting Items for Systematic Reviews and Meta-Analyses (PRISMA) guidelines for a scoping review.

### 4.2. Research Strategy

The literature search was performed in March 2024 and then repeated in November 2024 using PubMed, PubMed Central, BioMed Central, ScienceDirect, Wiley, and EBSCOhost. These databases were searched using the following Boolean Search terms: (Functional properties OR properties) AND hydrogel* AND (“cellulose nanocrystals” OR nanocellulose OR nanowhiskers) AND (healthcare OR “health care applications” OR biomedicine OR biomedical OR “biomedical applications”). The screening process was conducted independently by two reviewers, and discrepancies were resolved through their consensus.

### 4.3. Eligibility Criteria

The inclusion criteria for this literature review were as follows:

- Primary research study design.
- Open access and/or open archive availability of the full-text article.
- Language is English (or translated into English).
- Publication type is a journal article.
- Healthcare or biomedicine application/s are proposed in the study.
- Hydrogels incorporated with cellulose nanocrystals or nanocellulose, or cellulose nanowhiskers.
- Utilisation of in vitro (cell lines) and/or in vivo (animal) biomedical models and/or human clinical trials to test the CNC-incorporated hydrogel prototypes.
- Studies using appropriate controls for CNC-based hydrogels (i.e., hydrogels with CNC compared with hydrogels without CNC).

Exclusion criteria were as follows:

- Not following a primary research study design.
- Investigation of only bacterial nanocellulose- or cellulose nanofiber-based hydrogels.
- Hydrogels were not tested using in vitro or in vivo biomedical models or in a human clinical trial.
- No healthcare or biomedicine application proposed.
- Lack of appropriate controls for CNC-based hydrogels (e.g., comparison of hydrogels based on various CNC concentrations instead of comparing against a hydrogel without CNC).

### 4.4. Study Selection, Data Extraction, and Analysis

All the retrieved records were exported to EndNote 20.5, and any duplicate records were automatically removed. The remaining records were uploaded to the Joanna Briggs Institute System for the Unified Management of the Assessment and Review of Information (JBI SUMARI), where the screening of the included studies was conducted in two stages by two authors. The title and abstract screening stage identified and included studies that aligned with the inclusion criteria based on the titles and abstracts of the articles. The articles included in this stage were further screened in the full-text screening stage, where the authors read the full-text articles to decide on the inclusion of those articles. Any conflicts regarding inclusion/exclusion during the two stages of screening were resolved by a third author. Data from the included studies were extracted and compiled on a Microsoft Excel spreadsheet (Version 2501) in Microsoft 365.

Restricting the inclusion criteria to open-access or open-archive articles may have excluded relevant proprietary studies, introducing selection bias and limiting representativeness. This criterion was applied to ensure transparency and reproducibility, but may affect comprehensiveness.

## Figures and Tables

**Table 1:** CNC-incorporated hydrogel characterisation and evaluation.

Reference	CNC Characterisation	Hydrogel Formulation	Physicochemical Properties/Characterisation	Biological Endpoints	Sterility, Endotoxin and Others
[18]	0–1.5% *w*/*v* CNC concentration; characterisation not reported	3% *w*/*v* chitosan and CNC bioink with β-glycerophosphate (BGP) and hydroxyethyl cellulose (HEC) as gelling agents	Logarithmic shear rate sweep, dynamic frequency sweep, and dynamic time sweep indicating increased viscosity; degree of swelling	Scanning electron microscopy (SEM); live/dead assay and Quant-iT PicoGreen dsDNA assay indicating no effect on cell viability or proliferation on pre-osteoblast cartilage-like cells (MC3T3-E1 cells); alkaline phosphatase (ALP) activity indicating promoted osteogenesis onset; von Kossa indicating increased calcium mineralisation; haematoxylin and eosin (H&E) staining	-
[19]	0 and 1.5% *w*/*w* CNC concentration; characterisation not reported	2% *w*/*v* chitosan and CNC thermogelling hydrogel with BGP and HEC as gelling agents	Logarithmic sweep; dynamic frequency sweep; dynamic time sweep	Laser scanning confocal microscopy; live/dead assay indicating increased proliferation and survival of pre-osteoblast cartilage-like cells (MC3T3-E1 cells); Quant-iT PicoGreen dsDNA assay; ALP activity indicating increased osteogenic differentiation; H&E staining, von Kossa	-
[20]	0.3% *w*/*v* CNC concentration; characterisation not reported	Sodium alginate, 0.3% *w*/*v* CNC and sericin interpenetrating network hydrogel with hydroxyapatite and D-glucono-δ-lactone as crosslinking agents	Swelling and degradability behaviour indicating reduced biodegradation; X-ray diffraction (XRD) analysis; thermal gravimetric analysis (TGA); compressive strength	Cell counting kit-8 (CCK-8) assay indicating support of pre-osteoblast cartilage-like cell survival and proliferation (MC3T3-E1 cells); ALP activity indicating biomineralisation capacity	-
[21]	1–15% *w*/*v* CNC concentration; XRD for CNC crystallinity index	10% *w*/*v* gelatin with methacrylic anhydride (MA) (GelMA), 2% *w*/*v* hyaluronic acid methacrylate (HAMA) and CNC hydrogel with lithium phenyl2,4,6-trimethylbenzoylphosphinate (LAP) and 400 nm UV light as crosslinking agents	Swelling behaviour; 1H-NMR spectroscopy; XRD; temperature sweep; cyclic compressions; scanning transmission electron microscope (STEM) and SEM	Optical and laser scanning confocal microscopy; live/dead assay indicating increased chondrogenic cell proliferation (ATDC5 cells)	Sterilisation of CNCs under UV light for 40 min before use
[22]	1% and 2% *w*/*v* CNC concentration; dynamic light scattering for CNC particle size measurements	1% *w*/*v* alginate and CNC hydrogel	Flow, frequency, amplitude, and time sweeps	SEM indicating maintenance of cell morphology; live/dead assay indicating increased chondrocyte viability (TC28a2 cells)	Autoclaving at 121 °C and 1.4 bar
[23]	Cellulose nanowhiskers present; Method A: 10 mg/mL CNW; Method B: 0–30% CNW; XRD for CNC crystallinity index; atomic force microscopy for CNW length and width measurements	Chitosan, pectin and CNW hydrogel	TGA; gel fraction test; water vapour permeability	Agar disc diffusion method indicating increased antibacterial activity against Staphylococcus aureus and Escherichia coli at 10% and 20% CNCs; resazurin cell viability assay indicating increased dermal fibroblast cells in the 10% CNC hydrogel	-
[24]	1.82% *w*/*v* enzymatically pretreated tunicate nanocellulose (ETC)	0.82% *w*/*v* alginate and ETC hydrogel	Time-resolved shear oscillation test; amplitude sweep test; compression test; macroscopic assessment of adipocyte distribution and retention	H&E staining indicating improved shape-preservation and even distribution of adipocytes; image analysis of graft histology sections using ImageJ software (version 1.53t; National Institutes of Health, Bethesda, MD, USA) indicating improved cell survival	-
[25]	0–10% nanocellulose concentration relative to the solid content of gelatin	Nanocellulose and nanocellulose extravascular stent with astragaloside IV (AS-IV) drug loading	Expansion analysis; TGA	SEM; live/dead assay indicating good human umbilical vein endothelial cell proliferation; immunohistochemistry analysis for CD206, CD68, and iNOS indicating good in vitro biocompatibility with local tissue; H&E staining; Masson staining, flow cytometry analysis; enzyme-linked immunosorbent assay (ELISA); RT-qPCR; immunohistochemistry, drug release rate indicating controlled drug release; colour Doppler ultrasonography; Western blotting; in vivo degradation indicating controlled biodegradation	-
[9]	0.2–2% TEMPO-modified nanocrystalline cellulose (mNCC)	7% methacrylated gelatin (MeGel) with mNCC (mNG) hydrogel	1H-NMR spectroscopy; dynamic mechanical thermal analysis (DMTA) indicating increased stiffness	Live/dead assay; cell spreading and metabolism indicating increased adipose-derived mesenchymal stem cell migration and metabolic activity; MTT cell proliferation assay; quantitative PCR; Quant-iT PicoGreen dsDNA assay; dimethylmethylene blue assay indicating increased glycosaminoglycan deposition, alcian blue stain; alizarin red staining; 4-(dimethylamino)benzaldehyde (DMAB) indicating decreased hydroxyproline content; myofibroblastic activation; chondrogenic and osteogenic differentiation indicating inhibited osteogenic differentiation of adipose-derived mesenchymal stem cells	UV sterilisation of all materials for at least one hour before use
[26]	0–10% CNC	Gelatin and CNC hydrogel with gamma-radiation crosslinking at 25 °C at a dose of 30 kGy	XRD for crystallinity measurement, rheological analysis; swelling degree; TGA	Agar diffusion method indicating good biocompatibility with slight toxicity towards fibroblast cells (L929 cells); riboflavin drug loading and release analysis indicating inverse proportionality between CNC content and drug loading and release properties	Agar diffusion assay for in vitro cytotoxicity assessment using 0.01% neutral red staining
[11]	2.5% and 5% wt CNC	Gelatinised starch with furfuryl isocyanate (S-FI) and CNC with water-soluble PEG-based tetramaleimide (TTMI) crosslinking	Frequency sweep test; swelling ratio	In vitro chloramphenicol drug release analysis indicating increased drug loading ability and controlled drug release; SEM; in vitro cell viability and proliferation using MTT assay indicating increased fibroblast viability (L929 cells)	-
[27]	0.5% *w*/*w* CNC	2% *w*/*v* chitosan and CNC thermogelling hydrogel with BGP and HEC as gelling agents	Dynamic sweep test; gelation kinetics indicating improved gelation kinetics; in vitro mass loss and degradation analysis indicating prolonged hydrogel degradation	SEM, live/dead assay indicating no significant difference in cell viability; in vitro therapeutic protein (TRAIL) release measured using an ELISA indicating controlled release of TRAIL; in vitro cell kill ability measured using CellTiter-Glo cell viability luminescent assay	-
[28]	0–2.5% wt CNC	2.5% wt quaternised cellulose and CNC hydrogel with BGP as gelling agent	Dynamic sweep test; in vitro degradation measured via mass loss method indicating a slow degradation rate	Transmission electron microscopy (TEM) indicating the gradual decrease in initially triggered inflammatory responses, in vitro doxorubicin drug release indicating controlled drug release; MTT cell viability assay	In vivo biocompatibility via H&E staining
[29]	0–250 µg/mL aldehyde-modified CNC (CCHO); rod-like shape CNCs with a length and diameter of 127 ± 47 nm and 4.2 ± 1.9 nm, respectively	Nanocarbon dots and CCHO hydrogel	Frequency sweep test; photothermal and photodynamic performance using infrared thermal imaging	TEM; atomic force microscopy (AFM); UV-vis spectroscopy, photoluminescence spectroscopy; X-ray photoelectron spectroscopy (XPS); electron spin resonance (ESR) spectroscopy; SEM; CCK-8 assay indicating high biosafety and negligible cytotoxicity towards B16F10 melanoma cells and HeLa cervical cancer cells; live/dead assay; H&E staining; terminal deoxynucleotidyl transferase-mediated nick-end labelling (TUNEL) staining; immunofluorescence staining of Ki-67	Haematological assessment
[30]	10% wt CNC; modified as magnetic CNC (MCNC); morphology characterisation using TEM, CNC nanoparticle alignment using SEM	3:1 ratio of 3-dimethyl (methacryloyloxyethyl) ammonium propanesulfonate (DMAPS, 95%): Methacrylic acid (MAA, 99%) and CNC/MCNC with N,N′-methylenebis(acrylamide) (BIS, 99%) as crosslinking agent and 2-hydroxy-2-methylpropiophenone as initiator	Strain sweep and dynamic frequency sweep tests; tensile testing indicating CNC as a nano-reinforcer; XRD; degradation analysis indicating controlled biodegradation	Live/dead assay and fluorescence microscopy for measuring cell proliferation indicating increased cell viability and proliferation; potential soft robotic application demonstration	UV radiation for two hours

**Table 2:** Biomedical models and assays for exploring the tissue engineering applications of CNC-based hydrogels.

Reference	Biomedical Model	Assay/Experiment	Aim of the Assay	Proposed Application(s)	Tested CNC Concentrations	Role of CNC
[18]	Clonal non-transformed newborn mouse calvaria MC3T3-E1 pre-osteoblast cell line	Live/dead assay using calcein AM/ethidium homodimer staining and laser scanning confocal microscopy	Biocompatibility assessed as cell viability at 24 h	Bone tissue engineering	0, 0.5, 1.5% *w*/*v*	High ALP activity with a quicker onset of osteogenesis at high CNC concentrations; greater mineral deposition within the scaffold; increased osteogenic markers; extensive extracellular matrix formation; promotion of osteogenic differentiation with accelerated early alkaline phosphatase activity; calcium mineralisation and collagen formation in the extracellular matrix.
		Quant-iT PicoGreen dsDNA assay	Cell proliferation at 3 and 7 days			
		ALP assay	Early stages of osteogenic differentiation (bone formation)			
		von Kossa staining	Calcium deposition and matrix mineralisation (bone formation)			
		haematoxylin and eosin (H&E) staining	Extent of collagen formation in the ECM			
[19]	Clonal non-transformed newborn mouse calvaria MC3T3-E1 pre-osteoblast cell line	Live/dead assay using calcein AM/ethidium homodimer staining and laser scanning confocal microscopy	Biocompatibility assessed as cell viability at 24 h		0, 1.5% *w*/*v*	Enhanced preosteoblast proliferation and sustained growth; elevated ALP activity indicating increased early osteogenesis; increased collagen production and calcium deposition; supported cell survival and differentiation, as well as matrix maturation and mineralisation.
		Quant-iT PicoGreen dsDNA assay	Cell proliferation at 7, 14 and 21 days			
		ALP assay	Early stages of osteogenic differentiation (bone formation)			
		von Kossa staining	Calcium deposition and matrix mineralisation at 7, 14 and 21 days (bone formation)			
		haematoxylin and eosin (H&E) staining	Extent of collagen formation in the ECM at 7, 14 and 21 days			
[20]	Clonal non-transformed newborn mouse calvaria MC3T3-E1 pre-osteoblast cell line	SEM imaging	Cell proliferation, adhesion and migration on the CNC-hydrogel at 2 days of culture		0.3% *w*/*v*	Enhanced molecular interactions; increased compressive strength; improved mechanical properties with stronger interface interactions between CNCs and fibre network structure; induce a filling effect; regulate swelling properties and expansion of hydrogels; develop larger apatite particle thickness on the surface; boost biomineralisation capability.
		CCK8 assay	Biocompatibility was assessed as cell viability and cell number changes (proliferation) at 2 and 7 days of culture on the CNC-hydrogel			
		ALP assay	Osteogenic differentiation at 7 days			
[21]	Chondrogenic mouse ATDC5 cell line	Live/dead assay using calcein AM/propidium iodide staining and laser scanning confocal microscopy	Biocompatibility assessed as cell viability and change in cell numbers (proliferation) at 1, 4 and 7 days	Cartilage tissue engineering	1, 5, 10, 15% *w*/*v*	Increase the sol–gel transition temperature; reinforce the intermolecular interactions within polymer networks; enhance compressive modulus; regulate the swelling ratio and polymer network density; develop a high roughness morphology.
[22]	Human-derived TC28a2 immortalised chondrocyte cell line	Live/dead assay using calcein AM/ethidium homodimer staining and fluorescence microscopy	Biocompatibility assessed as cell viability at 24 h		1, 2% *w*/*v*	Promoted matrix entanglement; increased storage modulus; strong shear-thinning properties; reduced cell sedimentation within the polymer matrix; increased cell viability.
		Fluorescence microscopy	Cell morphology and cell migration on CNC-hydrogel at 1 and 7 days			

**Table 3:** Biomedical model and assays used to assess the wound healing and skin repair capacities of CNC-based hydrogels.

Reference	Biomedical Model	Assay/Experiment	Aim of the Assay	Proposed Application	Tested CNC Concentrations	Role of CNC
[23]	Human dermal fibroblast primary cell line (HDFa)	Disc diffusion method	Antimicrobial activity after 24 h	Wound dressing (skin tissue engineering)	Method A: 1 mg/mL Method B: 4, 10, 20, 30%	To increase mechanical reinforcement; increase thermal stability; increase porosity; increase net water vapour transmission rate; weak/no cytotoxicity
		Resazurin assay	Biocompatibility was assessed as cell viability over 7 days			

**Table 4:** Biomedical models and assays used to evaluate CNC-based hydrogels as potential medical implants.

Reference	In Vitro Model	In Vivo Model	Experiment/Assay	Aim of the Investigation	Role of CNC-Based Hydrogel	Tested CNC Concentrations	Role of CNC
[24]	*-*	Balb/C mouse model	Macroscopic assessment of explanted hydrogel grafts after 30 days	Shape stability of grafts; shape and content of adipocyte retention; adipocyte distribution	Autologous fat grafting	1.82% *w*/*v*	To assist in shape and volume retention in grafts, resulting in the retention of more adipocytes
			haematoxylin and eosin (H&E) staining of core sections of explanted grafts	Qualitative assessment of adipocyte distribution, count and characterisation; mean adipocyte content and diameter; graft cross-sectional area			
[25]	HUVEC secondary endothelial cell line	-	Liquid chromatography mass spectrometry was conducted on PBS from the solution in which the astragaloside-IV-incorporated CNC–hydrogel stent was incubated in and on serum collected from blood samples obtained after the stent implantation	Chinese herbal anti-inflammatory medicine with autophagy induction potential, Astragaloside-IV; release rates at 1, 3, 5, 10, 20 and 30 days	Prevention of re-stenosis of the coronary stent (via inhibition of endothelial–mesenchymal transition	0, 5, 10%	Inhibited the expansion of the grafted vein; slowed in vivo degradation rate; similar macrophage levels to the control group, which indicates biocompatibility
			Comet assay	Biocompatibility assessed by measuring any DNA damage induced by the CNC hydrogel stent at 6, 12, 18 and 24 h			
			CCK8 assay	Cell viability and cell number changes (proliferation) at 24, 48 and 72 h			
			Live/dead assay using fluorescent dyes and fluorescence microscopy				
			Transmission electron microscopy	Effect of high and low doses of IL-1β on autophagosome and autophagolysosomal contents in EndMT cells			
			Bright field microscopy	Morphological changes in EndMT cells after AS-IV intervention			
	-	Mice	Immunohistochemistry analysis of local tissue for CD206, CD68, and iNOS	Local tissue compatibility of stents			
			haematoxylin and eosin (H&E) staining and Masson staining of the kidney, heart, liver, spleen and lung	Damage to important organs induced by stents			
			Flow cytometry of blood and spleen for lymphocytes, CD45, CD3, CD45R, CD11B+ F4/80+ and CD11B+ after 3 and 7 days	Pro- and anti-inflammatory gene expression responses induced by stents			
			ELISA for IL-1B, IL-2, IL-6, IL-4, IL-10, IL-13 of blood samples after 3 and 7 days				
			RT-PCR of spleen samples for IL-1B, IL-2, IL-6, IL-4, IL-10 and IL-3 after 3 and 7 days				
		Sprague-Dawley rats	Colour Doppler detection	Blood flow through the grafted vein and its patency rate			
			haematoxylin and eosin (H&E) staining after 4 weeks	Intimal thickness; vessel wall thickness; intimal area			
			Western blotting for α-SMA, CD31, vimentin, slug, snail, twist, slug, LC3II, p62, beclin 1, AMPK, p-AMPK, mTOR, p-mTOR on vein grafts	Potential of AS-IV to inhibit the EndMT process through the autophagy pathway			
			RT-qPCR of IL-1β, IL-2, IL-6, IL-4, IL-10, and IL-13 on spleen tissue				
			RT-qPCR of IL1β, TGF-β, CD31, α-SMA, slug, snail 1, twist, vimentin, beclin1, LC3II, p62, AMPK, and mTOR on a section of vein graft				
[9]	Human adipose-derived mesenchymal stem cell line	-	MTT assay	Biocompatibility is assessed as cell viability, morphology and migration	Cardiac valve regeneration	0.2, 0.4, 0.8, 1.5, 2.0%	To increase cell spreading and metabolic activity over time; increase cell differentiation; increase strain energy, transition strain, elastic modulus, and initial strain modulus; inhibit osteogenic differentiation
			Live/dead assay using calcein AM/ethidium homodimer staining and confocal microscopy				
			Quant-iT PicoGreen dsDNA assay; dimethyl methylene blue (DMMB) assay/Alcian blue staining; 4-(dimethylamino)benzaldehyde (DMAB) assay	ECM deposition via measuring dsDNA, sulphated glycosaminoglycans (sGAGs) and hydroxyproline contents, respectively			
			α-SMA, vimentin, MMP1 and MMP2 expression	Myofibroblastic activation			
			ACAN and Sox9 expression	Chondrogenic/spongiosa differentiation (cardiac valve regeneration)			
			α-SMA, vimentin, Rnx2, OCN and OPN expression	Osteogenic potential in osteogenic growth media conditions			
			Alizarin red staining	Osteogenic differentiation in growth and osteogenic growth media			

**Table 5:** Models and experiments used to assess the drug delivery applications of CNC-based hydrogels.

Reference	In Vitro Model	In Vivo Model	Experiment/Assay	Aim of the Investigation	Role of CNC-Based Hydrogel	Tested CNC Concentrations	Role of CNC
[26]	Mouse fibroblast L929 secondary cell line	-	Agarose diffusion assay	Cytotoxicity based on cell layer changes and/or zone of cell inhibition	Platform for controlled small intestine-related drug delivery of riboflavin	0, 4, 8, 10%	Good biocompatibility with slight toxicity; inverse proportionality between CNC content and drug loading and release
[11]	Mouse fibroblast L929 secondary cell line	-	MTT assay	Biocompatibility is assessed by cell viability and proliferation	Antimicrobial drug, chloramphenicol delivery	2.5, 5 wt%	Improved cell viability and drug loading; controlled drug release
[27]	Therapeutic C17 mouse neural stem cells (iNSCs)	-	Live/dead assay using calcein AM/ethidium homodimer staining and laser scanning confocal microscopy	Biocompatibility assessed as stem cell viability on 1, 7, 14 and 30 days	Antitumour drug, tumour necrosis factor-α (TNF-α)-related apoptosis-inducing ligand (TRAIL) proapoptotic protein, delivery	0.5% *w*/*w*	Maintenance of cell viability; slow hydrogel degradation rate; reinforcing agent; improve gelation kinetics; assist sustained release of cells from hydrogels; controlled drug release
	Human U87 glioblastoma multiforme tumour cells		CellTiter-Glo^®^ cell viability luminescent assay	Tumour cell killing ability was assessed as tumour cell viability at 24 and 72 h			
[28]	African green monkey kidney fibroblast COS-7 secondary cell line	-	MTT assay	Biocompatibility assessed as cell viability at 24 h	Antitumour drug, doxorubicin, delivery	0, 1, 1.5, 2.5 wt%	Gradually diminished the initially triggered inflammatory responses; no evidence of necrosis, haemorrhaging, oedema or muscle damage; improved dimensional stability; slow degradation rate; controlled drug release
	-	C57 mice with subcutaneous injections of H22 cancer cells into the left forelimb armpit	Tumour volume; haematoxylin and eosin (H&E) staining of tumour, heart, liver and spleen	Antitumour activity of hydrogel			
[29]	Mouse melanoma B16F10 primary cell line and human cervical cancer HeLa primary cell line	-	CCK8 assay; live/dead assay using calcein AM/propidium iodide staining and fluorescence microscopy	Tumour cell killing ability is assessed as tumour cell viability	Antitumour drug delivery platform using nanocarbon dots	1.5 wt%	High biosafety and negligible cytotoxicity; tumour-killing ability through simultaneous photothermal therapy and photodynamic therapy
			2,7-dichlorodihydro-fluorescien diacetate (DCFH-DA) staining as a radical oxygen species (ROS) indicator and confocal laser scanning microscopy	Intracellular ROS generation; photodynamic therapy (PDT) effect			
	-	B16F10 tumour-bearing nude mice	haematoxylin and eosin (H&E) staining of heart, lung, liver, kidney and spleen	Pathological changes induced by hydrogels			
			Haematological assessments: whole blood tests and blood biochemical analysis	In vivo toxicity of hydrogels			
			Haematoxylin and eosin (H&E), terminal deoxynucleotidyl transferase-mediated nick-end labelling (TUNEL), and Ki-67 staining of tumour slices	Antitumour performance of hydrogels			
[30]	Mouse embryonic fibroblast NIH-3T3 secondary cells	-	Live/dead assay using calcein AM/ethidium homodimer staining and confocal microscopy	Cell viability and proliferation on 1, 3 and 5 days	Soft robot in controlled biomolecule delivery	10 wt%	Magnetic navigation of the soft robot using superparamagnetic behaviour

## Data Availability

The raw data that support the findings of this review are available from the corresponding author upon reasonable request. The checklist is available in Supplementary (File S1).

## Supplementary Materials

The following supporting information can be downloaded at: https://www.mdpi.com/article/10.3390/gels12030207/s1, File S1: The ARRIVE guidelines 2.0: author checklist.
